# The roles of grouper clathrin light chains in regulating the infection of a novel marine DNA virus, Singapore grouper iridovirus

**DOI:** 10.1038/s41598-019-51725-5

**Published:** 2019-10-30

**Authors:** Liqun Wang, Qiang Li, Songwei Ni, Youhua Huang, Jingguang Wei, Jiaxin Liu, Yepin Yu, Shaowen Wang, Qiwei Qin

**Affiliations:** 1 College of Marine Sciences, South China Agricultural University, Guangzhou, 510642 China; 2 College of Oceanology and meteorology, Guangdong Ocean University, Zhanjiang, 524088 China; 3 College of Veterinary Medicine, South China Agricultural University, Guangzhou, 510642 China; 4 Laboratory for Marine Biology and Biotechnology, Qingdao National Laboratory for Marine Science and Technology, Qingdao, 266000 China

**Keywords:** Biological fluorescence, Microbiology, Marine biology

## Abstract

Clathrins, composed of clathrin heavy chains (CHCs) and clathrin light chains (CLCs), are usually hijacked by viruses for infection. However, the role of CLCs, especially in regulating fish virus infection, remains poorly understood. Here, two isoforms of CLCs were cloned from the red-spotted grouper (*Epinephelus akaara*) (EaCLCa and EaCLCb). Both EaCLC transcripts were expressed in all examined tissues, and the expression of EaCLCa was much higher than that of EaCLCb. Over-expressing EaCLCa-W119R mutant significantly reduced Singapore grouper iridovirus (SGIV) infectivity. However, no effect of EaCLCb-W122R on SGIV infection was observed. The detailed steps were further studied, mainly including virus attachment, entry and the following transport to early endosomes. EaCLCa-W119R mutant notably inhibited internalization of SGIV particles with no effect on SGIV attachment. Furthermore, EaCLCa-W119R mutant obviously impaired the delivery of SGIV to early endosomes after virus internalization. In addition, the EaCLCa-W119R mutant markedly reduced the colocalization of SGIV and actin. However, EaCLCb is not required for such events during SGIV infection. Taken together, these results demonstrate for the first time that EaCLCa and EaCLCb exerted different impacts on iridovirus infection, providing a better understanding of the mechanisms of SGIV infection and opportunities for the design of new antiviral strategies.

## Introduction

Clathrin-coated vesicles (CCVs) are major carriers for cargo trafficking at the plasma membrane, the trans-Golgi network (TGN), and the endosomal system and often carry proteins, lipids and even pathogens^1^. The best-characterized unit of clathrin consists of three clathrin heavy chains (CHCs) and three clathrin light chains (CLCs) which are unstructured until they binding to CHCs. Whereas CHCs provide the structural backbone of CCVs, the functional role of CLCs is poorly defined. CLCs are reported to suppress spontaneous CHC self-assembly *in vitro* and thus enable the control of cellular clathrin assembly by adaptor and regulatory proteins^2–4^. However, evidence regarding the necessity of CLCs for the endocytosis of different cargos remains contradictory. Knockdown of either or both CLCs by siRNA in mammalian cells has no measurable effect on the clathrin-mediated endocytosis of transferrin, epidermal growth factor, low density lipoprotein receptor or cation-independent mannose-6 phosphate receptor (CI-MPR)^5,6^. However, the uptake of G-protein-coupled receptors does depend on CLCs^7^. Another important role of CLCs is to regulate clathrin-mediated trafficking between the TGN and the endosomal system by acting as recruitment proteins for huntingtin-interacting protein 1-related (HIP1R), enabling HIP1R to regulate the interactions of clathrin-coated structures with the actin cytoskeleton^6^.

The viral life cycle depends heavily on cellular factors for virus attachment, entry, replication, assembly, and progeny virus release. To date, clathrin is the factor most commonly used for pathogen internalization into the host cell. Numerous viruses, such as influenza virus, African swine fever virus and bovine ephemeral fever virus, hijack clathrin-mediated endocytosis as the primary means of entry^8–10^. Moreover, some viruses, such as vesicular stomatitis virus, human papillomavirus type 16, and adenovirus, require both actin and clathrin for entry^11–13^. In addition, recent studies have shown that clathrin also affects other events in viral life cycles. A functional clathrin-binding motif within the large antigen protein (Ag-L) of hepatitis delta virus (HDV) was identified, and the interaction between clathrin and Ag-L significantly affected HDV assembly^14,15^. The nonstructural protein mammalian reovirus (MRV) could recruit cellular clathrin to viral factories, further disrupting normal clathrin-dependent trafficking^16^. However, most studies focus on the interactions of clathrin with viruses, and the role of CLCs in virus infection remain largely unknown.

There are two isoforms of CLCs in all metazoans, CLCa and CLCb, encoded by different genes. They share approximately 60% protein sequence identity and are expressed at characteristically different levels in all tissues. Their longest shared fragment is 22 residues near the N terminus, starting with three negatively charged residues (EED), termed the consensus (CON) sequence, which serves as a binding site for huntingtin interacting proteins (HIPs) and HIP1-related (HIP1R) and regulates clathrin self-assembly^4,17,18^. In mammals, from the N terminus to the C terminus, other features shared by the two CLCs include an EF-hand that is responsible for binding to calcium (Ca^2+^), the heavy-chain-binding region (HC), neuronally expressed inserts (N), and a calmodulin-binding domain (CBD). The unique regions in CLCa and CLCb are Hsc70 and serine phosphorylation sites (P), respectively^1^. To date, we know little about the different functions of CLCa and CLCb, especially during the viral life cycle.

Iridoviruses, large dsDNA viruses, have attracted increasing attention due to the threat they pose to aquaculture and biodiversity^19^. To date, iridoviruses can infect invertebrates and poikilothermic vertebrates, including fish, amphibians, and reptiles^19,20^. The type species of the genus *Ranavirus*, frog virus 3 (FV3), uses clathrin mediated endocytosis (CME) for entry mammalian cells^21^. Singapore grouper iridovirus (SGIV), which causes a serious and highly lethal systemic disease in grouper aquaculture, is a novel member of the genus *Ranavirus* and the family *Iridoviridae*^22^. Our previous study showed that SGIV could enter host cells through CME and then undergo transport to the endosomal compartment^23^. However, the impact of clathrin, especially CLCs, on the life cycle of SGIV still remains limited.

In this study, EaCLCa and EaCLCb, derived from grouper *Epinephelus akaara*, were cloned and characterized. Consistent with previous results^23^, EaCLC mainly affect early events during SGIV infection. Notably, although EaCLCa and EaCLCb shared approximately 65% sequence identity, they showed clearly different effects on SGIV infection. Data from the dominant-negative EaCLCa mutant demonstrated that EaCLCa significantly affects SGIV entry and the subsequent step, transport to the early endosomes, possibly by regulating the interaction of virus and actin. In contrast, EaCLCb showed no measurable effect on SGIV infection.

## Results

### Sequence analysis of EaCLC

**Figure 1:**
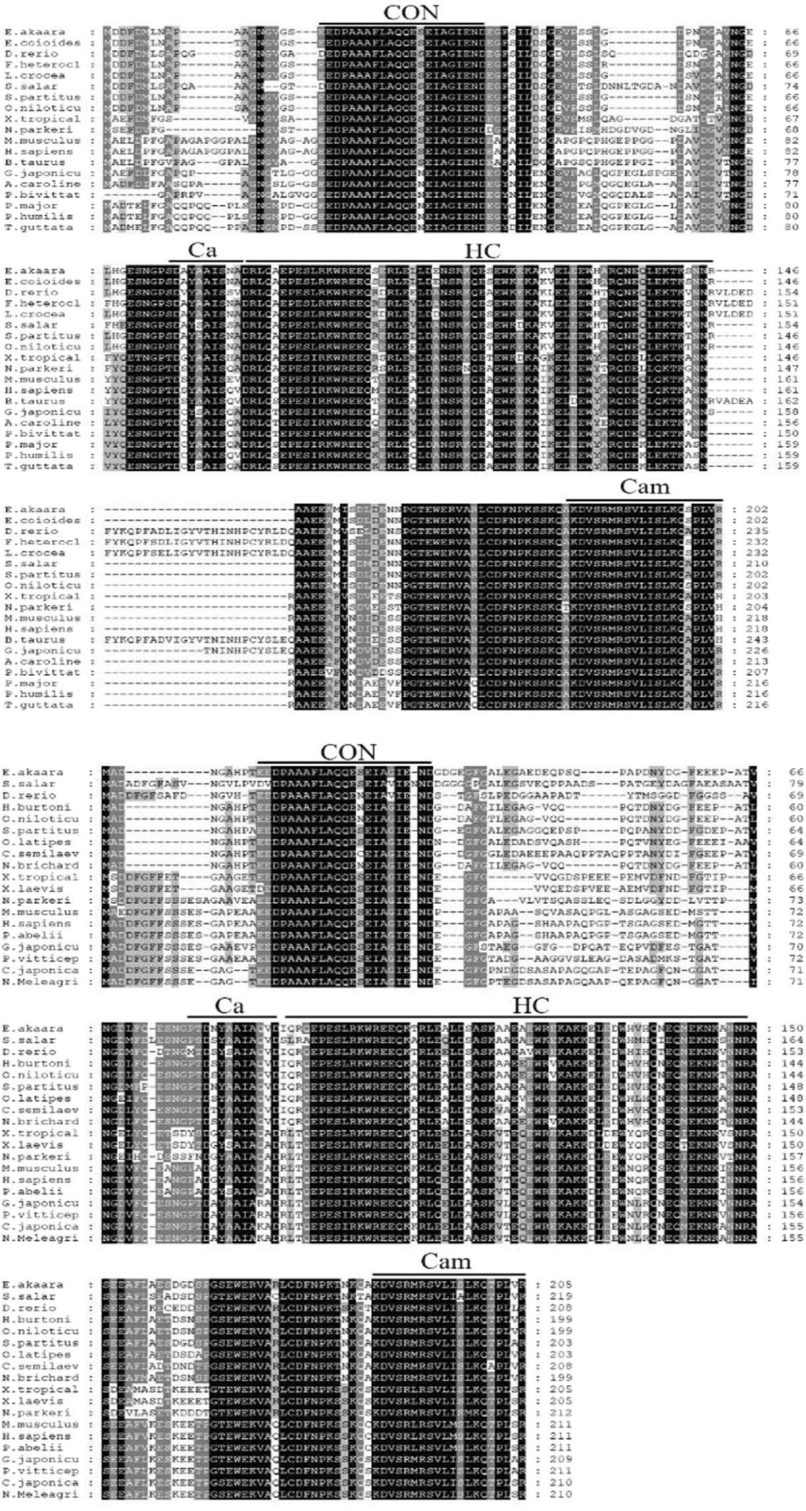
Multiple sequence alignment of EaCLC from different species. (**A,B**) Amino acid sequence alignments of EaCLCa (**A**) and EaCLCb (**B**). CON, the 22-residue consensus sequence shared by CLCa and CLCb; Ca, calcium-binding site; HC, the heavy-chain-binding region; Cam, calmodulin-binding domain.

Based on the transcriptome analysis, we obtained the full-length open reading frames of EaCLCa and EaCLCb by PCR amplification. EaCLCa encodes a 202-amino-acid protein and has remarkably high sequence conservation in vertebrates according to the alignment of amino acid sequences 99% and 75% identity to *Epinephelus coioides* and *Homo sapiens*, respectively (Fig. 1A). EaCLCb encodes a 205-amino-acid protein with a greater sequence divergence 92% and 68% identity to *Stegastes partitus* and *H.sapiens*, respectively (Fig. 1B). Amino acid alignment showed that both EaCLC contain CON, Ca, HC and Cam domains. EaCLCa shares 65% identity with EaCLCb. Phylogenetic analysis indicated that EaCLCa and EaCLCb are both sorted into the Osteichthyes branch, which is separate from amphibian and mammals (Supplementary Fig. S1).

### Expression pattern of EaCLC

**Figure 2:**
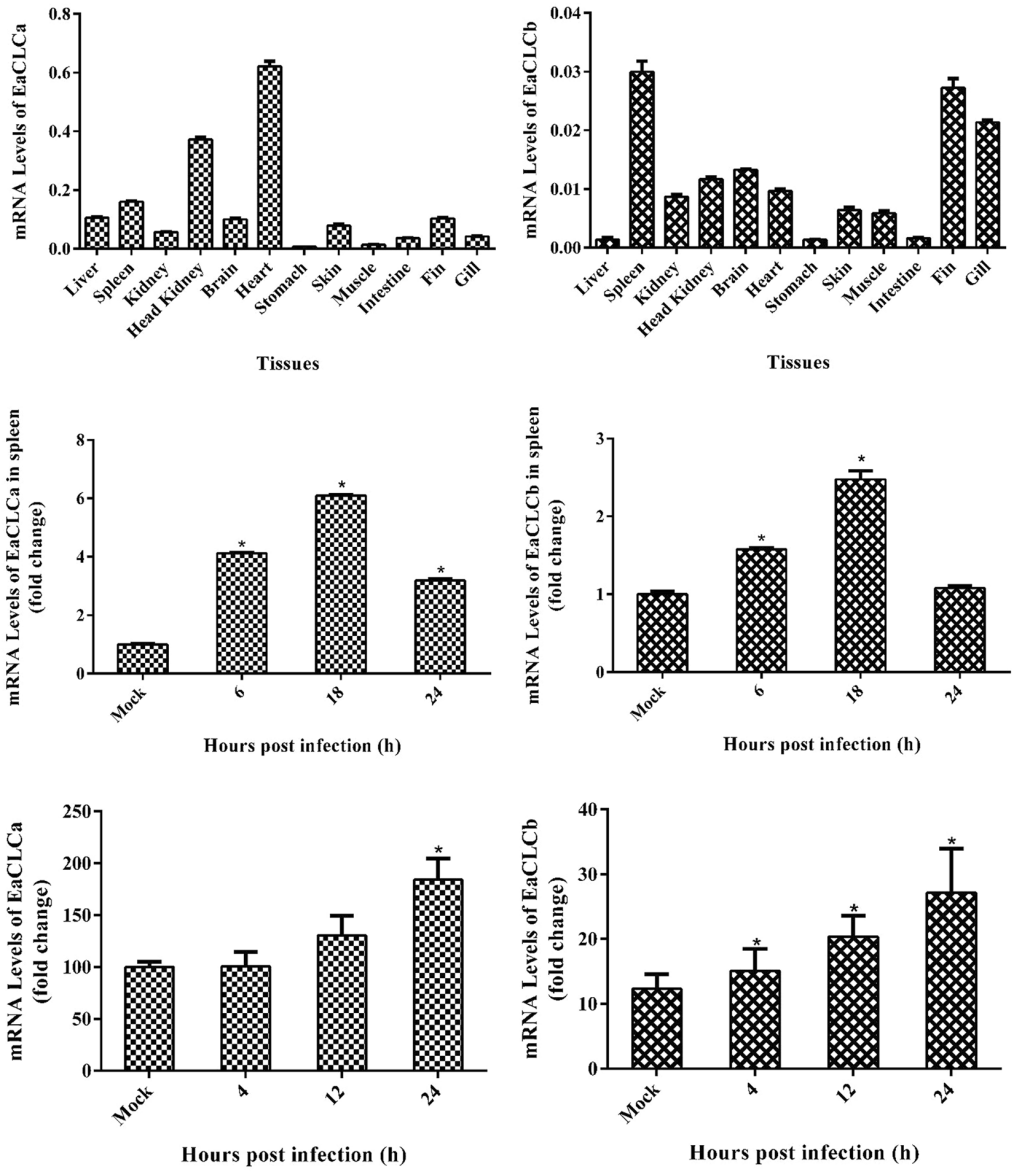
mRNA Levels of EaCLC quantified by qRT-PCR. (**A**,**B**) The mRNA levels of EaCLCa (**A**) and EaCLCb (**B**) in different tissues from healthy grouper. (**C**,**D**) The mRNA levels of EaCLCa (**C**) and EaCLCb (**D**) in the *E. akaara* infected with SGIV. (**E**,**F**) The mRNA levels of EaCLCa (**E**) and EaCLCb (**F**) in GS cells after infection with SGIV. The data were tested using qRT-PCR and are indicated as the mean ± SEM (n = 4). Statistic differences are shown as *p < 0.05.

Both EaCLC were found to be distributed in all tissues examined in grouper, *E. akaara*, but at characteristically different levels. EaCLCa was predominantly detected in the heart and head kidney, followed by the spleen, liver, fin, brain, skin, kidney, gill, intestine, muscle and stomach (Fig. 2A). However, EaCLCb was highly expressed in the spleen, fin and gill but showed low expression in the liver, stomach and intestine (Fig. 2B). Interestingly, the expression of EaCLCa was much higher than that of EaCLCb in all tissues. To evaluate the expression changes of EaCLCa and EaCLCb in response to SGIV, the grouper was challenged with SGIV and the transcript of EaCLCa and EaCLCb was detected using qRT-PCR. Our results showed that the expression level of EaCLCa and EaCLCb firstly upregulated at 6 h, and reached the peak at 18 h, then decreased since 24 h. In addition, the expression of EaCLCa was also higher than that of EaCLCb in infected spleen (Fig. 2C,D). Besides, we further explore the expression profiles of EaCLCa and EaCLCb upon virus infection in GS cells. As shown in Fig. 2E,F, the transcription levels of both EaCLCa and EaCLCb were clearly upregulated.

### Subcellular localization of EaCLC

**Figure 3:**
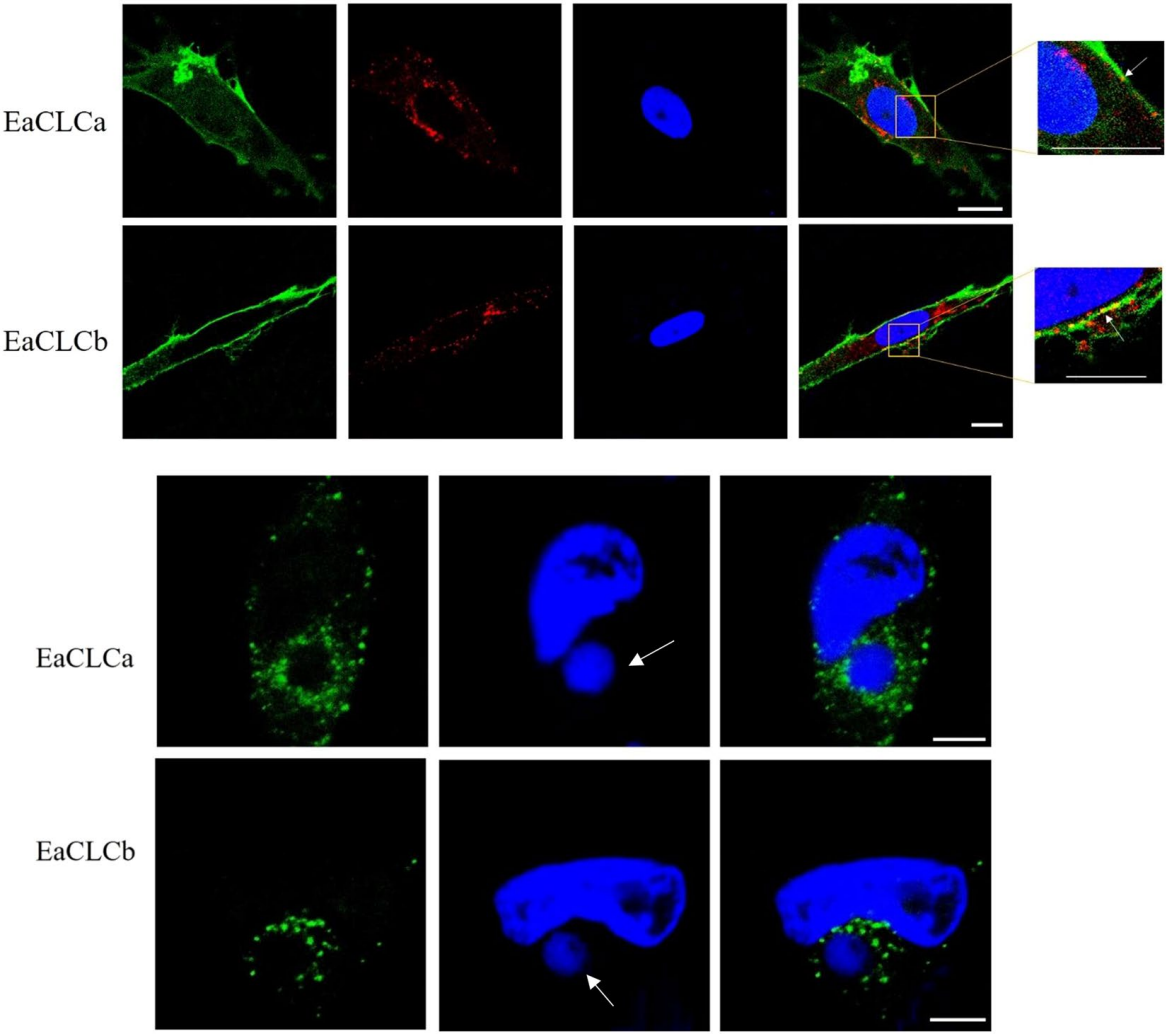
Subcellular distribution of EaCLC. (**A**) Distribution pattern of EaCLC. GS cells were transfected separately with pEGFP-EaCLCa and pEGFP-EaCLCb and then stained with Hoechst 33342 and DiD to indicate the nucleus (blue) and cell membrane (green). The arrows show the EaCLC located on the cell membrane. Scale bars represent 10 μm. (**B**) EaCLC located near the virus factory. GS cells transfected with pEGFP-CLCs were infected with SGIV and fixed at 16 hpi. The nucleus and virus factory were stained by Hoechst 33342 (blue). The arrow shows the virus factory. Scale bars represent 5 μm.

The intracellular localization of EaCLC in living cells was investigated. Both EaCLCa and EaCLCb displayed punctate structures located in the cytoplasm and on the cell membrane (Fig. 3A). After infection with SGIV, EaCLC were observed to surround the virus factory (Fig. 3B), preliminarily proving their role in virus replication and assembly.

In addition, we detected the distribution of EaCLCs and SGIV at different times post-infection. Upon infection, Alex-Fluor 647 labelled SGIV particles rapidly colocalized with EaCLCa and EaCLCb (Supplementary Fig. S2A). Moreover, the percentage of SGIV particles that colocalized with EaCLCa was slightly higher than the percentage that colocalized with EaCLCb (Supplementary Fig. S2B).

### Effect of EaCLC on SGIV infectivity

It is reported that the residue 130 W in HC domain of CLCa and 127 W in HC domain of CLCb are essential for their interaction with CHC^24^. To further analyse the potential role of EaCLC in SGIV infection, we generated two mutations of EaCLC to perturb the normal function of CLC. A point mutation in the residue 119 of EaCLCa and residue 122 of EaCLCb, from W to R, were performed. In the cells transfected with pEGFP-EaCLCa-W119R or pEGFP-EaCLCb-W122R, the GFP-EaCLC mutant remained soluble and was hardly observed as fluorescent spots (Supplementary Fig. S3).

**Figure 4:**
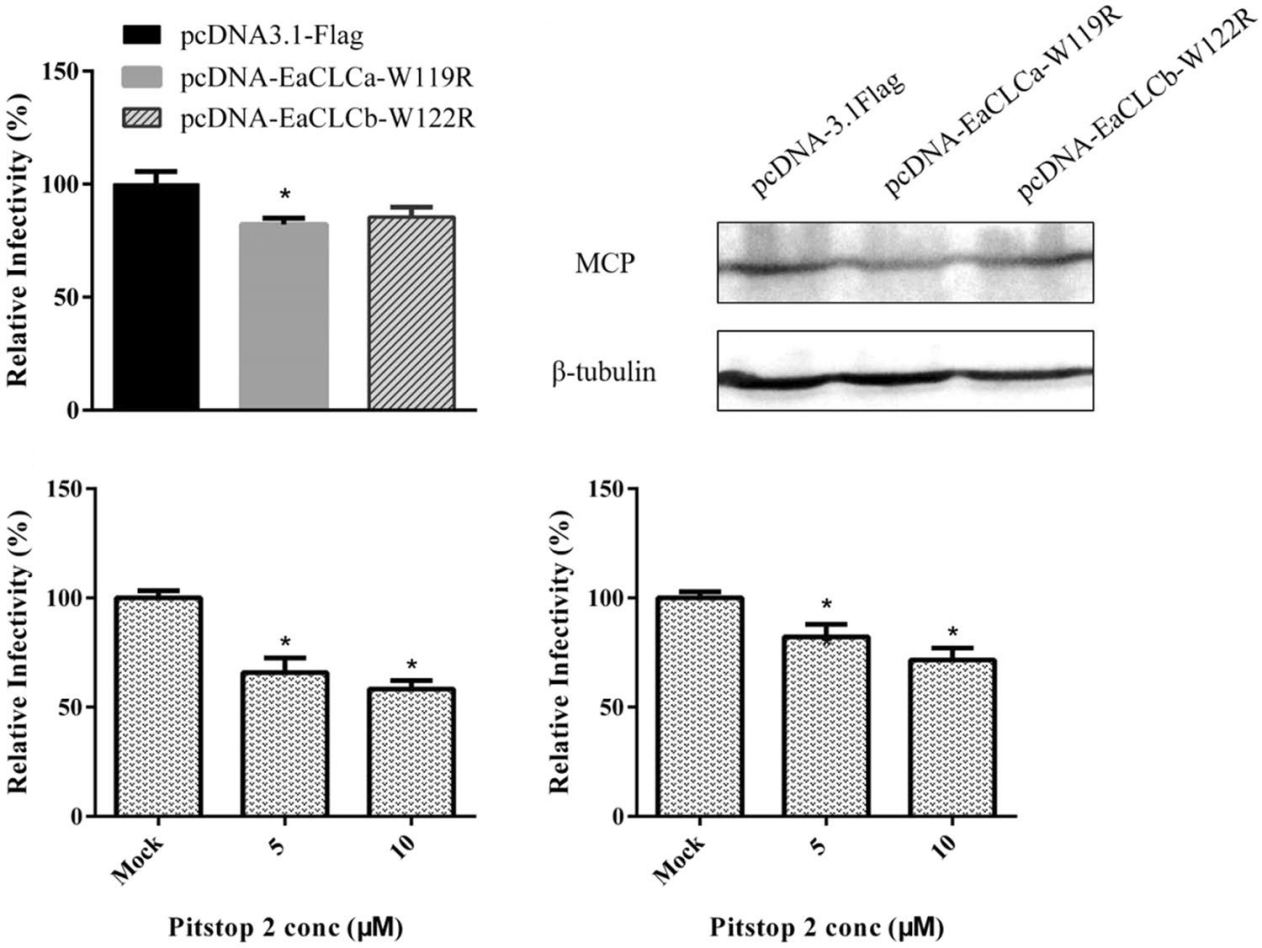
SGIV infection is dependent on EaCLCa and EaCHC. (**A**) Disruption of EaCLCa inhibits SGIV infection. GS cells transfected with pcDNA3.1-flag, pcDNA-EaCLCa-W119R, and pcDNA-EaCLCb-W122R were incubated with SGIV 24 h after transfection. At 24 hpi, the cells were fixed and stained with Hoechst 33342 to indicate the nucleus and virus factory. The infectivity was quantified by calculating the percentage of virus factory to the cells. The infectivity was arbitrarily set as 100%. The data shown are the means and the standard error of the mean (SEM). *p < 0.05. (**B**) Viral protein level after transfection with EaCLCa-W119R and EaCLCb-W122R. GS cells transfected with pcDNA3.1-flag, pcDNA-EaCLCa-W119R, and pcDNA-EaCLCb-W122R, respectively. After 24 h, cells were harvested for western blot, and β-tubulin was used as the internal control. (C-D) Treatment with Pitstop 2 pre-infection (**C**) or post-infection (**D**) block SGIV infection. GS cells were either pre-treated with different concentrations of Pitstop 2 for 2 h or treated with Pitstop 2 at 2 hpi. Subsequently, the cells were incubated with SGIV for another 24 h and then fixed for quantification. The data are shown as the means ± SEM. *p < 0.05.

We assessed the effect of EaCLC on SGIV infection by counting the infected cells. GS cells were transfected separately with pcDNA-3.1, pcDNA-EaCLCa-W119R and pcDNA-EaCLCb-W122R, incubated with SGIV and processed to stain the viral factory. Only EaCLCa mutant overexpression significantly decreased the percentage of SGIV-infected cells (Fig. 4A). Besides, overexpression of EaCLCa mutant also decreased the protein levels of SGIV MCP (Fig. 4B) and the severity of cytopathic effect (CPE) (Supplementary Fig. S4). The suppression effects of EaCLCa mutant was similar with the results obtained using Pitstop 2, a small-molecule drug that specifically disrupts the terminal domain of CHC (Fig. 4C). Additionally, when added at the post entry step, Pitstop 2 could still impair SGIV infection, implying that CHC may play a role in virus replication, assembly and release (Fig. 4D). However, whether EaCLCa could influence such events requires further analysis. Taken together, these results show that EaCLCa plays an important role in SGIV infection.

### EaCLCa is required for SGIV entry

**Figure 5:**
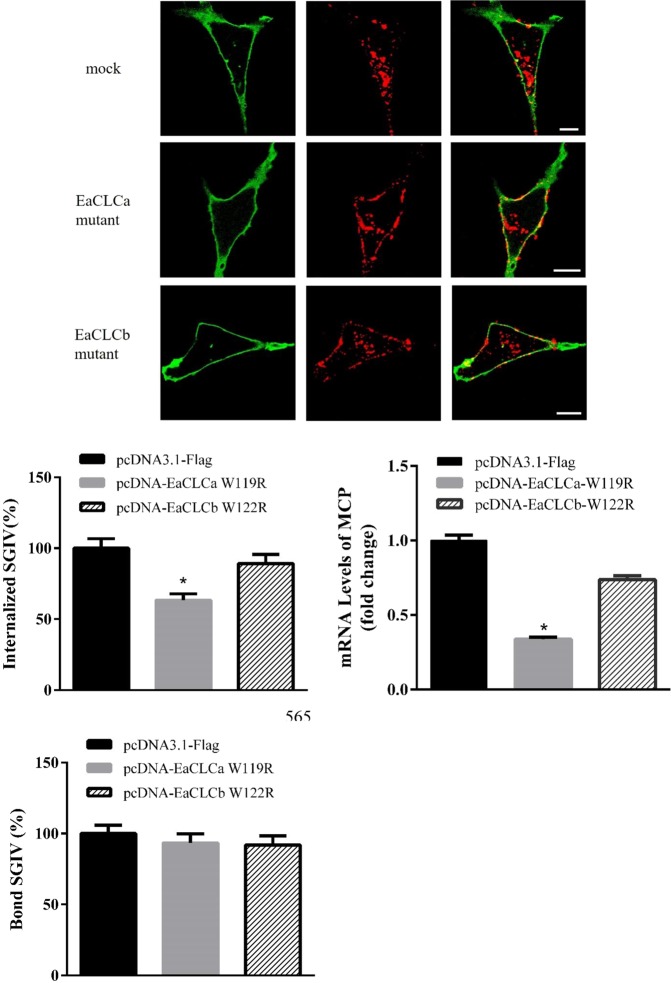
EaCLCa is essential for SGIV entry. (**A**) EaCLCa clearly impairs SGIV uptake. GS cells transfected with pcDNA3.1-flag, pcDNA-EaCLCa-W119R and pcDNA-EaCLCb-W122R were incubated with Alex-Fluor 647 labelled SGIV at 4 °C for 30 min, and then the temperature was changed to 28 °C to stimulate infection. At 2 hpi, the cells were washed twice with medium and fixed with paraformaldehyde. The samples were stained with DiO to show the cell boundaries (green). Scale bars represent 10 μm. (**B**) Quantification of internalized SGIV particles. More than 90 cells were randomly selected and analysed by a MATLAB program. The internalized SGIV was quantified as the percentage of treated cells with internalized viruses relative to for the value in mock cells. The internalized SGIV of mock cells was arbitrarily set to 100%. The data are shown as the mean ± SEM. (**C**) Qverexpression of EaCLCa mutant decreased SGIV transcription. mRNA levels of MCP were determined by qRT-PCR. GS cells transfected with pcDNA3.1-flag, pcDNA-EaCLCa-W119R and pcDNA-EaCLCb-W122R were incubated with SGIV at 4 °C for 1 h, and then the temperature was shifted to 28 °C. At 4 hpi, the cells were collected for RNA extraction and qRT-PCR analysis. (**D**) Quantification of SGIV particles still bound on the cell membrane. More than 30 cells were randomly selected and analysed by the MATLAB program. The bound SGIV was quantified as the percentage of treated cells with viruses attached on the cell membrane relative to that for mock cells. The bound SGIV of mock cells was arbitrarily set as 100%. The data are shown as the means ± SEM. *p < 0.05.

To test our hypothesis that EaCLC are involved in SGIV entry, we measured single SGIV particle entry events in GS cells transfected with dominant-negative EaCLC. GS cells were transfected separately with pcDNA-3.1, pcDNA-EaCLCa-W119R and pcDNA-EaCLCb-W122R, incubated with Alex-Fluor 647 labelled SGIV for 2 h and processed for confocal imaging. Visually, a noticeable reduction in virus uptake was observed in cells expressing EaCLCa mutants compared with that in cells expressing pcDNA-3.1 (Fig. 5A). Moreover, as shown in Fig. 5B, SGIV entry was significantly inhibited (up to 40% inhibition) in the presence of the EaCLCa mutant. In addition, EaCLCa mutant overexpression inhibited the viral gene transcription (Fig. 5C). However, SGIV internalization was unaffected by the EaCLCb mutant (Fig. 5B). These results revealed that EaCLCa was involved in SGIV entry, but EaCLCb had no significant effect.

### EaCLCa affects SGIV entry at a post-binding step

**Figure 6:**
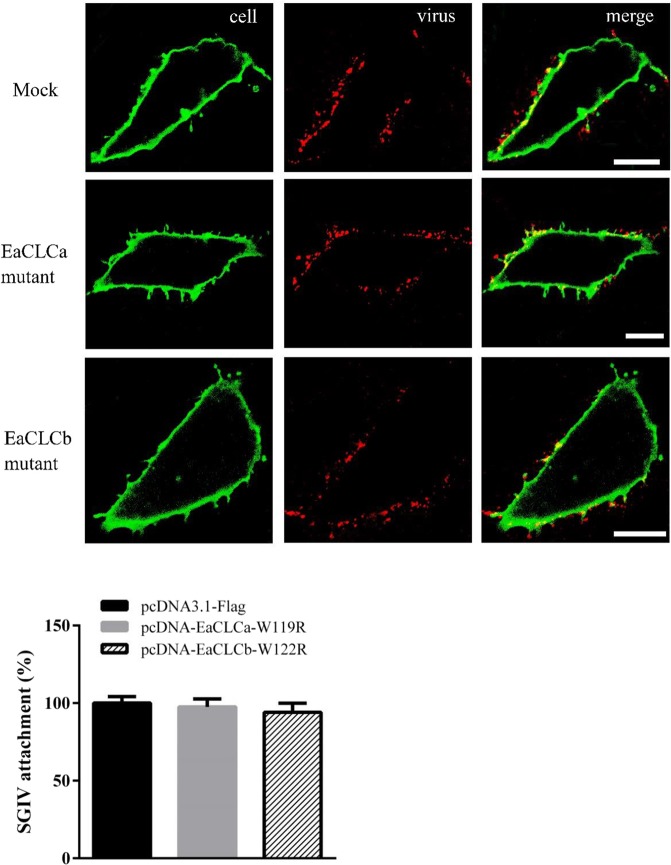
EaCLCa affects SGIV attachment. (**A**) Confocal images of SGIV attachment after different treatments. GS cells transfected with pcDNA3.1-flag, pcDNA-EaCLCa-W119R and pcDNA-EaCLCb-W122R were incubated with Alex-Fluor 647 labelled SGIV at 4 °C for 1 h and then immediately fixed with paraformaldehyde. The samples were stained with DiO to show the cell boundaries (green). Scale bars indicate 10 μm. (**B**) Quantification of SGIV particle binding on the cell membrane. More than 30 cells were randomly selected and analysed by a MATLAB program. Each experiment was repeated three times. The SGIV attachment was quantified as the percentage of treated cells with virus particles attached on the cell membrane relative to that for mock cells. SGIV attachment to mock cells was arbitrarily set as 100%. The data are shown as the means ± SEM. *p < 0.05.

To further probe the role of the EaCLC in SGIV entry, a virus attachment assay was performed. The cells expressing EaCLC mutants were incubated with SGIV at 4 °C for 1 h, followed by the removal of unbound virus, immediate fixation, fluorescent imaging and the quantification of cell-bound SGIV by a MATLAB program. As shown in Fig. 6, there was no significant variance between EaCLC mutants and mock cells. In addition, the number of SGIV particles attached on the cell surface remained unchanged during SGIV entry (Fig. 5D). These data suggested that the EaCLC had no effect on virus attachment.

### EaCLCa mutant disrupts SGIV particle trafficking to the early endosome

After internalization by clathrin-mediated endocytosis, cargo molecules, including receptors, ions, lipids, and even viruses, are usually transported to early endosomes (EEs) for further sorting.

**Figure 7:**
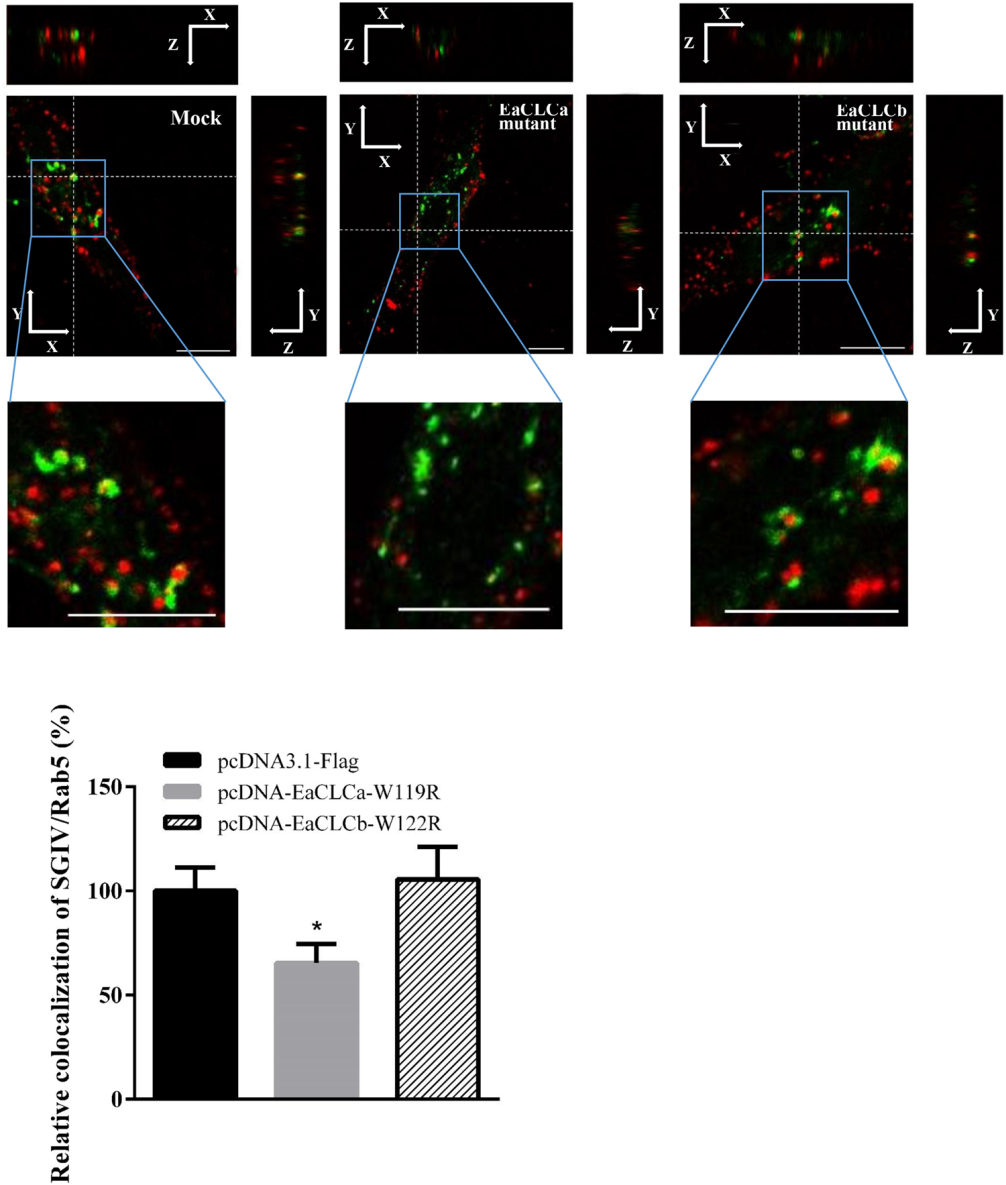
EaCLCa affects the colocalization of SGIV particles with Rab5. (**A**) Detection of the colocalization of SGIV and Rab5 after different treatments in 3D images. GS cells co-transfected pEGFP-Rab5 (green) with pcDNA3.1-flag, pcDNA-EaCLCa-W119R, and pcDNA-EaCLCb-W122R, respectively, were incubated with Alex-Fluor 647 labelled SGIV (red) at 4 °C for 20 min and then immediately transferred to 28 °C to initiate infection. The cells were fixed at 0.5 hpi, respectively. Scale bars represent 10 μm. (**B**) Quantification analysis of SGIV colocalized with Rab5 in 2D images. Using the MATLAB program, the colocalization of SGIV and Rab5 was quantified as the percentage of virus particles colocalized with Rab5 relative to the total virus internalized in the cell. The colocalization of SGIV and Rab5 in mock cells was arbitrarily set as 100%. The data are shown as the means ± SEM. *p < 0.05.

To explore whether EaCLC affect the transport of SGIV particles to EEs, the colocalization of SGIV particles to Rab5 (marker of EEs) was analysed. In EaCLC mutants and pEGFP-Rab5 co-transfected cells, EGFP-Rab5 also showed a punctate cytoplasmic distribution. However, at 0.5 hpi, the colocalization between SGIV particles and EEs was significantly decreased in cells overexpressing mutant EaCLCa compared with the mock cells (Fig. 7). However, EaCLCb had no obvious effect on the transport of SGIV particles to EEs (Fig. 7). In addition, disruption of EaCLCa did not appear to alter the morphology and distribution of Rab5-positive endosomes. Taken together, these results indicated that the regulation of SGIV infection by EaCLCa also occurred at the post internalization step.

### EaCLCa mutant alters the colocalization between SGIV and actin filaments

**Figure 8:**
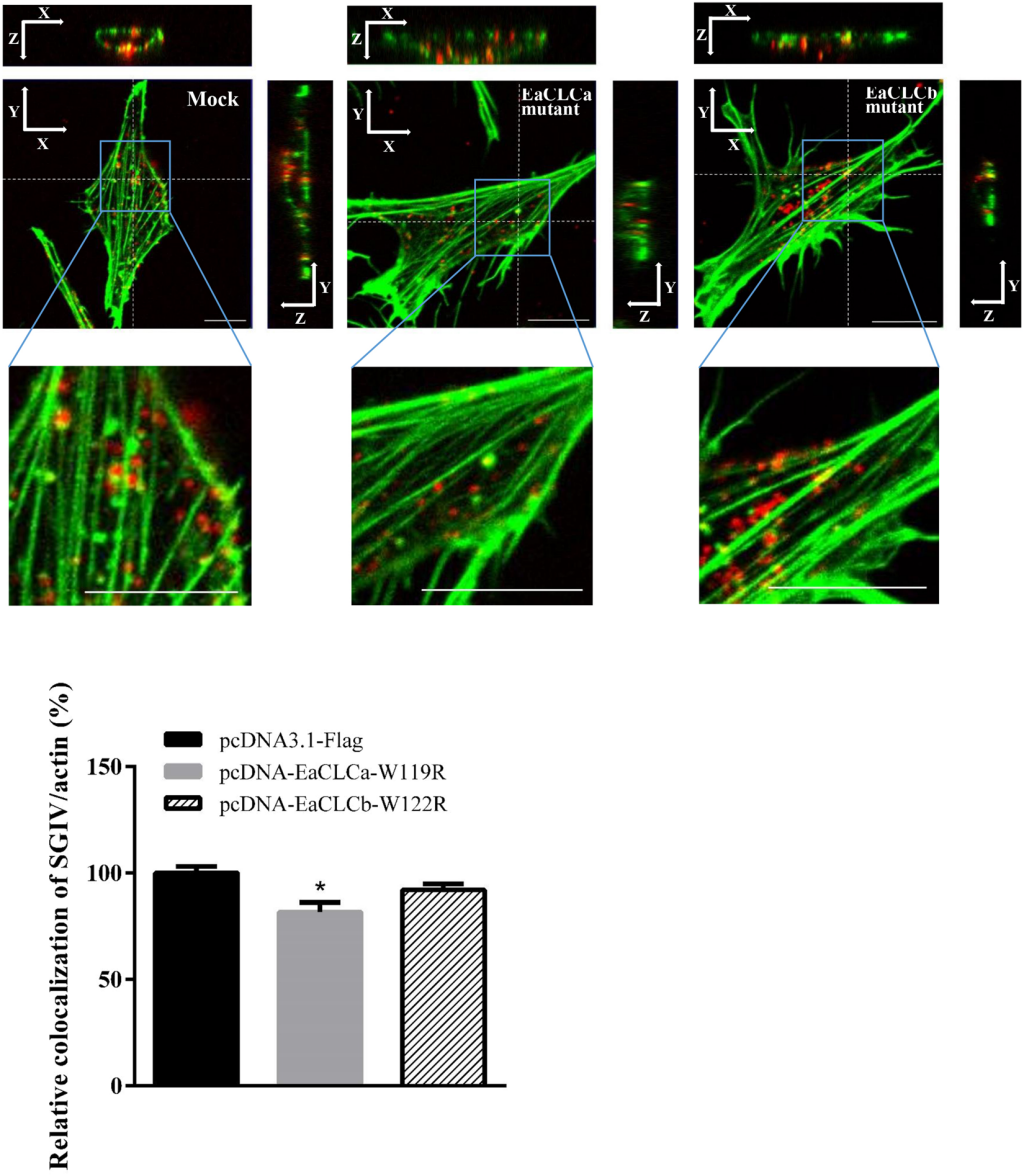
EaCLCa alters the colocalization of SGIV particles with actin filaments. (**A**) Detection of the colocalization of SGIV and actin after different treatments in 3D images. GS cells transfected with pcDNA3.1-flag, pcDNA-EaCLCa-W119R, and pcDNA-EaCLCb-W122R, respectively, were incubated with Alex-Fluor 647 labelled SGIV (red) at 4 °C for 20 min and then immediately transferred to 28 °C to initiate infection. The cells were fixed at 1 hpi and stained for actin using Alexa Fluor 488 phalloidin (green) for 30 min. Scale bars represent 10 μm. (**B**) Quantification analysis of SGIV colocalization with actin in 2D images. The colocalization of SGIV and actin was quantified by Pearson’s colocalization coefficient. The colocalization of SGIV and actin in mock cells was arbitrarily set as 100%. The data are shown as the means ± SEM. *p < 0.05.

Some viruses, such as vesicular stomatitis virus (VSV) depend on actin filaments for entry^25^. VSV enter the cells by CME assisted by actin filaments^25^. Meanwhile, Many reports have demonstrated that CLCs can regulate actin assembly^1,6,26,27^. Therefore, we detected correlations between SGIV particles and actin in different treatments to explore the effect of EaCLC on the interaction between actin and SGIV. Alexa Fluor 488 phalloidin was used to label actin filaments. In cells transfected with pcDNA-EaCLCa-W119R, the actin distribution showed no obvious change, but the colocalization between SGIV particles and actin was significantly reduced compared with the mock cells (Fig. 8). However, there was no difference in the cells transfected with the EaCLCb mutant. These results revealed that EaCLCa could affect the interaction between SGIV particles and actin filaments.

## Discussion

Clathrin is the principal structural component of clathrin-coated carriers and is involved in a wide range of cellular processes as well as the life cycle of viruses. The structural unit of clathrin, the triskelion, consists of trimerized CHCs and three CLCs. To date, only a single CLC has been identified in yeast and invertebrates. However, in vertebrates, two forms of CLC (CLCa and CLCb) appear. Previous reports indicated that two CLC isoforms arose by local gene duplication and contributed to the development of increasing complexity during chordate evolution^28^. In mammals, CLCa and CLCb exhibit distinct levels of expression in different tissues, with CLCa and CLCb dominant in the lymphoid tissue and the brain, respectively. In groupers, EaCLCa and EaCLCb were both distributed in all tissues analysed in our study, but they were predominantly detected in different tissues. These different characteristics of CLCs in different species may imply a potential for diverse functions.

GFP-tagged EaCLC appeared as spots in the cytoplasm and cell membrane, displaying the typical pattern observed in mammalian cells. In addition, EaCLCa and EaCLCb showed similar localization in the cell. In mammalian cells, CLCa and CLCb distribute randomly in cellular triskelia, such that all four types of triskelia (aaa, aab, bba, and bbb) are always present^29^.

Notably, the disruption of EaCLCa and EaCLCb by dominant-negative interference had different effects on SGIV entry. Only the EaCLCa mutant significantly impaired SGIV uptake, verifying that EaCLCa was critical for SGIV entry. Unexpectedly, although EaCLCb shared high sequence similarity and a similar distribution with EaCLCa, it had no significant effect on SGIV entry, suggesting functional differences between the EaCLC in regulating SGIV entry. Thus far, this report is the first on the different functions of CLC in iridovirus infection. Conformational variation in CLC may result in functional differences. In addition, attachment assays showed that neither of the EaCLC affected the binding of SGIV particles to the cell membrane, demonstrating that EaCLCa affects a post binding step of SGIV entry, which is consistent with the role of EaCLCa in cellular membrane trafficking.

Prompted by the previous finding that SGIV was delivered to early endosomes after internalization, we demonstrated here that EaCLCa mutant significantly reduced the localization of SGIV particles to EEs, suggesting that EaCLCa also affects the step after virus entry. In addition, by calculating SGIV-infected cells, we found that SGIV infectivity was also significantly inhibited only when EaCLCa was disrupted. However, because of technological limitations, it remains unclear whether EaCLCa could affect other steps, such as virus replication and assembly. In contrast, by adding the small molecular inhibitor Pitstop 2 at different times, we found that EaCHC affected both virus entry and the steps after virus internalization. EaCLC may have similar effects on events after virus entry. During the late stage of SGIV infection, EaCLC tightly surrounded the viral factories, suggesting that clathrin may be recruited for virus replication, assembly or release. A few viruses have been reported to be directly connected with clathrin during virus assembly. Ag-L of hepatitis delta virus (HDV) was demonstrated to be related to CHC, thus affecting HDV particle assembly^14,15^. μNS of mammalian reovirus (MRV) not only recruits clathrin to viral factories but also interferes with normal functions of clathrin in cellular membrane trafficking^16^.

In addition, the overexpression of mutant EaCLCa significantly impaired the colocalization between SGIV particles and actin filaments, suggesting that EaCLCa probably affects virus entry by influencing the interaction between SGIV and actin. However, the EaCLCb mutant had no effect. In mammals, CLCs have been reported to recruit Hip1 and Hip1R (referred to as Hip1/R) to form the LC-Hip1/R-actin complex, which mainly regulates actin assembly on clathrin-coated structures^26,27^. A dramatic change in actin organization could be observed in CLC knockdown cells. However, the disruption of EaCLCa or EaCLCb did not visibly change the morphology of actin. The data showed that EaCLCa probably affected the colocalization of SGIV with actin by other means.

Based on this study, we propose that EaCLCa and EaCLCb have different roles in SGIV infection. EaCLCa obviously regulates distinct steps in the SGIV life cycle, including virus entry and transport to early endosomes or actin filaments. Surprisingly, EaCLCb had no influence on these events during SGIV infection. Further studies are needed to explore the detailed mechanisms involved in the different roles of EaCLC in virus infection.

## Methods

### Fish, cells and viruses

Red-spotted groupers (*E. akaara*) were purchased from a marine-culture farm in Hainan Province. The animal study was carried out in accordance with the recommendations of Guangdong Medical Laboratory Animal Centre. The protocol was approved by the South China Sea Institute of Oceanology, Chinese Academy of Sciences.

The grouper spleen (GS) cell line used in this study was maintained in our laboratory^30^. GS cells were cultivated in Leibovitz’s L-15 medium containing 10% foetal bovine serum (Gibco) at 28 °C. The SGIV used in this study was originally isolated from diseased grouper (*Epinephelus tauvina*)^22^. SGIV was propagated in GS cells, and virus stocks were maintained at −80 °C.

### Identification and sequence analysis of CLCs from *E. akaara* (EaCLC)

Based on the EST sequences from the grouper transcriptome^31^, the full-length open reading frames of EaCLC were amplified using the primers listed in Table 1. The putative amino acid sequence of EaCLC was analysed by BioEdit, the Expasy search program (http://au.exasy.org/tools) and BLAST in NCBI (http://www.ncbi.nlm.nih.gov/blast). The domain structure of the EaCLC was predicted by SMART (http://smart.embl-heidelberg.de/). Multiple sequence alignment and phylogenetic analysis of EaCLC were carried out by using Clustalx 1.83 (http://www.ebi.ac.uk/clustalW/) and MEGA 4.0 software (http://megasoftware.net/), respectively.

**Table 1:** Sequence of primers used in this study.

Primers	Sequence (5′-3′)
pcDNA-EaCLCa-F	GGGGTACCGAATGGATGATTTTGAC
pcDNA-EaCLCa-R	CGGAATTCCTAACGGACTAGCG
pcDNA-EaCLCb-F	GGGGTACCGAATGGCTGACAA
pcDNA-EaCLCb-R	CGGAATTCCTAGCGCACTAGAG
pEGFP- EaCLCa-F	GGGTACCATGGATGATTTTGACATGCTGA
pEGFP- EaCLCa-R	CGGATCCCTAACGGACTAGCGGGGACT
pEGFP- EaCLCb-F	GGAAGATCTATGGCTGACAACGGCGCA
pEGFP- EaCLCb-R	CGGGGTACCCTAGCGCACTAGAGGTGTCTGTTTG
pEGFP- EaRab5-F	GGGGTACCATGGCAAGTAGAAGTGGAGC
pEGFP- EaRab5-R	CGGGATCCTCAGGAAGCCAAGGAGCCCGAT
RT- EaCLCa-F	GGAGGGAGGAGCAAAGTG
RT- EaCLCa-R	GGTTGAAGTCGCAGAGCC
RT- EaCLCb-F	CGAACAGCCGTCTCAAC
RT- EaCLCb-R	TGTCTTCTGCTCCTCCCT
RT- actin-F	TACGAGCTGCCTGACGGACA
RT- actin-R	GGCTGTGATCTCCTTCTGCA
pDsRed1- EaCLCa-F	GGGTACCATGGATGATTTTGACATGCTGA
pDsRed1- EaCLCa-R	CGGATCCCTAACGGACTAGCGGGGACT
pDsRed1- EaCLCb-F	GGAAGATCTATGGCTGACAACGGCGCA
pDsRed1- EaCLCb-R	CGGGGTACCCTAGCGCACTAGAGGTGTCTGTTTG
pcDNA-EaCLCa mutant-F	GAGTCAGAGCGGAAGGAGAAAGCCAAGGTGGAGCTGGAAG
pcDNA-EaCLCa mutant-R	TTTCTCCTTCCGCTCTGACTCCTGCTTGCGAGAATTTTC
pcDNA-EaCLCb mutant-F	GAGGCAGAGCGGAGAGAGAAAGCCAAAAAGGAGCTGGAGGAC
pcDNA-EaCLCb mutant-R	TTTCTCTCTCCGCTCTGCCTCTGCTGCCTTGGATGCTGAGTC

The underlined text means enzyme digestion sites (pcDNA-EaCLCa-F, pcDNA-EaCLCa-R, pcDNA-EaCLCb-F, pcDNA-EaCLCb-R, pEGFP-EaCLCa-F, pEGFP-EaCLCa-R, pEGFP-EaCLCb-F, pEGFP-EaCLCb-R, pEGFP-Rab5-F, pEGFP-Rab5-R, pDsRed1-EaCLCa-F, pDsRed1- EaCLCa-R, pDsRed1-EaCLCb-F, pDsRed1-EaCLCb-R) and mutant sites (pcDNA-EaCLCa mutant-F, pcDNA-EaCLCa mutant-R, pcDNA-EaCLCb mutant-F, pcDNA-EaCLCb mutant-R) added in the designing primers.

### Reagents and plasmid construction

Hoechst 33342 and Pitstop 2 were purchased from Sigma-Aldrich. Pitstop 2 were dissolved in dimethyl sulfoxide (DMSO) according to the manufacturer’s instructions. The lipophilic dyes DiO and DiD were purchased from Biotium. The fluorescent dyes Alexa Fluor 647 and Alexa Fluor 488 phalloidin were purchased from Invitrogen. anti-β-tubulin was purchased from Abcam (USA). peroxidase-conjugated affinipure goat anti-rabbit IgG were purchased from proteintech (USA).

Using the primers listed in Table 1, the full-length CLCs were constructed in vectors including pcDNA3.1-flag, pEGFP-N3, and pmDsRed-C1 (Invitrogen). Site-directed mutants, including EaCLCa-W119R and EaCLCb-W122R, were all subcloned into the pEGFP-N3, pmDsRed-C1 and pcDNA3.1-flag vectors using specific primers (Table 1) and the Fast Mutagenesis Kit V2 (Vazyme). Tryptophan (W)119 of CLCa and W122 of CLCb were both replaced with arginine(R). In addition, the pEGFP-Rab5 vector was maintained in our laboratory. The constructed plasmids were confirmed by sequencing.

### Expression patterns of EaCLC

Total RNA was extracted from different tissues of healthy groupers, including the head kidney, kidney, liver, spleen, intestine, stomach, gill, brain, heart, skin, and muscle, and then examined by qRT-PCR.

For viral infection *in vitro*, GS cells were infected with SGIV at a multiplicity of infection (MOI) = 1 and collected at 0, 4, 12, and 24 hpi for further qRT-PCR analysis.

For viral infection *in vivo*, groupers were injected with 200 μl SGIV (2 × 10^4^ TCID_50_/ml), and collected at different time points (0, 6, 18, 24 h). At indicated time points, spleen was collected for further qRT-PCR analysis.

Total RNA was extracted from grouper tissues or GS cells using the SV Total RNA Isolation System (Promega) according to the manufacturer’s protocol, examined by electrophoresis and then reverse transcribed into cDNA by the cDNA synthesis kit Rever Tra Ace (TOYOBO, Japan). qRT-PCR was carried out using a Light Cycler 480 Real-time PCR system (Roche, Basel, Switzerland) with SYBR Green as the fluorescent dye, according to the manufacturer’s protocol (TOYOBO). β-Actin was used as an internal control. All primer pairs are listed in Table 1. Each assay was carried out in triplicate using the following cycling conditions: 94 °C for 5 min, followed by 45 cycles of 5 s at 94 °C, 10 s at 60 °C and 15 s at 72 °C. The data were calculated as fold changes based on the transcription levels of the targeted genes normalized to β-actin and given in terms of relative mRNA transcription level as the means ± standard deviation.

### Virus purification and fluorescence labelling

Purified and fluorescence-labelled SGIV particles were produced as described previously^22,23^. Briefly, SGIV was inoculated onto monolayers of GS cells at an MOI of approximately 0.1. When the cytopathic effect was sufficient, SGIV particles were harvested by repeated freeze-thaw cycles, cleared of cell debris at 12,000 × g (Beckman Allegra X-15R Centrifuge) for 30 min at 4 °C, and the supernatant pelleted by ultracentrifugation at 200,000 g (Beckman 70 Ti rotor) for 1 h at 4 °C. Subsequently, the virus pellet was resuspended in TN buffer (50 mM Tris-HCl, 150 mM NaCl, pH 7.5) and further purified with a sucrose gradient (30–60%, wt/vol) at 150,000 g (Beckman SW 40 rotor) for 1 h at 4 °C. The viral band was washed with TN buffer, repelled by ultracentrifugation at 100,000 × g for 1 h at 4 °C, and then resuspended in TN buffer and stored at −80 °C until use.

SGIV particles were labelled with fluorescent dye by incubation with Alexa Fluor 647 in phosphate-buffered saline (PBS) (pH 7.4) at room temperature for 2 h with gentle vortexing for Alexa Fluor 647 dye conjugating to the primary amines (R-NH_2_) of proteins on viral capsid. Then, unincorporated dye was removed by three high-speed centrifugations at 14,000 × g (Beckman Micrifuge 20 R Centrifuge) at 4 °C for 60 min. Labelled virus was examined under a transmission electron microscope (Supplementary Fig. S5) and stored at 4 °C. To test whether labelling Alexa Fluor 647 would affect the infectivity of SGIV, we used the western blot to investigate the protein level of SGIV MCP (Supplementary Fig. S6).

### Cell transfection

GS cells grown to 50 or 70% confluence in 35-mm glass bottom culture dishes or 24-well plates were transiently transfected with each plasmid using Lipofectamine 2000 (Invitrogen) according to the manufacturer’s instructions. Briefly, 0.8 μg of each plasmid and 2 μl of Lipofectamine were diluted in 100 μl of serum-free medium containing OptiMEM and L-15 medium (1:1). After a 30-min incubation, the DNA-liposome mixture was added to the cells for 6 h of incubation at 28 °C. Then, the cells were replaced in serum-containing medium and cultured for further analysis.

### Virus infection assay

GS cells were cultured in 24-well plates, transfected with pcDNA-3.1, pcDNA-EaCLCa-W119R and pcDNA-EaCLCb-W122R, and then incubated with SGIV (MOI = 1) 24 h after transfection. At 24 hpi, the cells were collected for western blot or fixed with 4% paraformaldehyde. The fixed samples would be stained with Hoechst 33342 to highlight the nucleus and viral factory. Images were taken by an inverted fluorescence microscope (Zeiss, Germany), and the infected foci were counted.

### SGIV binding and entry assays

The virion binding and uptake assay was performed by measuring the amounts of SGIV particles attached to the cell surface and in the cytoplasm, respectively. In brief, 24 h after plasmid (pcDNA-3.1, pcDNA-EaCLCa-W119R or pcDNA-EaCLCb-W122R) transfection, GS cells were incubated with Alex-Fluor 647 labelled SGIV (MOI = 10) at 4 °C for 1 h to allow virus binding. Then, the cells were either fixed immediately with 4% paraformaldehyde or transferred to 28 °C to allow SGIV entry and fixed at 2 hpi. These samples were further analysed by Confocal Laser Scanning Microscope (CLSM). The experiments repeated three times independently.

### Colocalization analysis of SGIV particles and Rab5 or actin

GS cells were transfected with different combined sets of plasmids (pEGFP-Rab5 and pcDNA-3.1, pEGFP-Rab5 and pcDNA-EaCLCa-W119R, pEGFP-Rab5 and pcDNA-EaCLCb-W122R). At 24 h post transfection, the cells were incubated with Alex-Fluor 647 labelled SGIV (MOI = 10) at 4 °C for 20 min to allow virus binding, then transferred to 28 °C to allow SGIV entry and fixed at 0.5 hpi. Further analysis was performed by CLSM.

The plasmids pcDNA-3.1, pcDNA-EaCLCa-W119R and pcDNA-EaCLCb-W122R were separately transfected into cells 24 h prior to SGIV infection. Alex-Fluor 647 labelled SGIV (MOI = 10) was added to the cells at 4 °C for 20 min, and the cells were then moved to 28 °C to allow SGIV entry and fixed at 1 hpi. The fixed samples were stained with Alexa Fluor 488 phalloidin to highlight actin. Further analysis was performed by CLSM.

### Confocal imaging assay

Fluorescent images were obtained through a ZEISS LSM 7 DUO confocal microscope. The signals of EGFP and DiO were excited using a 488-nm Ar-Kr laser and a 500–550 nm bandpass filter for emission. Alexa 647 and mCherry were excited with a 633-nm helium neon laser and a 650–700 nm bandpass filter for emission. Fluorescence emission was collected and imaged through a 100× (numerical aperture, 1.4) oil immersion objective. Image stacks were acquired with a 200-nm z-step size. For quantification analysis, confocal images were obtained by noise filtering, edge detection and fluorescent signal extraction using a MATLAB program. Approximately 100 cells were randomly analysed by the MATLAB program to calculate the number of SGIV particles on the cell membrane or in the cytoplasm. In addition, the 3D images of the colocalization between virus particles and Rab5/actin were performed by CLSM. Furthermore, the quantification of the colocalization between SGIV and Rab5/actin were analysed in more than 60 randomly chosen cells (2D images) by a MATLAB program.

### Western blot

Cells were harvested and dissolved in RIPA buffer. Proteins were separated by 10% SDS-PAGE and transferred onto Immobilon-Polyvinylidene difluoride membranes (Millipore, Temecula, CA, USA). Blots were incubated with the indicated primary antibody: anti-β-tubulin (1:2,000 dilution), anti-SGIV major capsid protein (MCP) (1:1,000 dilution). Subsequently, they were incubated with peroxidase-conjugated affinipure goat anti-rabbit IgG (1:5,000 dilution). The polyclonal anti-MCP antibody of SGIV were prepared in our lab. Immunoreactive proteins were visualized using an Enhanced HRP-DAB Chromogenic Substrate Kit (Tiangen, China).

### Statistical analysis

Statistical significance was calculated using the Student’s test (*p < 0.05).

### Ethics statements

All animal-involving experiments of this study were approved by the Animal Care and Use Committee of College of Marine Sciences, South China Agricultural University, and all efforts were made to minimize suffering.

## Supplementary information

Supplementary Information

## References

[1] Brodsky FM (2012). Diversity of clathrin function: new tricks for an old protein. Annu. Rev. Cell Dev. Biol..

[2] Ungewickell E, Ungewickell H (1991). Bovine brain clathrin light chains impede heavy chain assembly *in vitro*. J. Biol. Chem..

[3] Ybe JA (1998). Clathrin self-assembly is regulated by three light-chain residues controlling the formation of critical salt bridges. Embo J..

[4] Chen CY, Brodsky FM (2005). Huntingtin-interacting protein 1 (Hip1) and Hip1-related protein (Hip1R) bind the conserved sequence of clathrin light chains and thereby influence clathrin assembly *in vitro* and actin distribution *in vivo*. J. Biol. Chem..

[5] Huang F, Khvorova A, Marshall W, Sorkin A (2004). Analysis of clathrin-mediated endocytosis of epidermal growth factor receptor by RNA interference. J. Biol. Chem..

[6] Poupon V (2008). Clathrin light chains function in mannose phosphate receptor trafficking via regulation of actin assembly. Proc. Natl. Acad. Sci. USA.

[7] Ferreira F (2012). Endocytosis of G protein-coupled receptors is regulated by clathrin light chain phosphorylation. Curr. Biol..

[8] Rust MJ, Lakadamyali M, Zhang F, Zhuang X (2004). Assembly of endocytic machinery around individual influenza viruses during viral entry. Nat. Struct. Mol. Biol..

[9] Hernaez B, Alonso C (2010). Dynamin- and clathrin-dependent endocytosis in African swine fever virus entry. J. Virol..

[10] Cheng CY (2012). Bovine ephemeral fever virus uses a clathrin-mediated and dynamin 2-dependent endocytosis pathway thatrequires Rab5 and Rab7 as well as microtubules. J. Virol..

[11] Cureton DK, Massol RH, Whelan SP, Kirchhausen T (2010). The length of vesicular stomatitis virus particles dictates a need for actin assembly during clathrin-dependent endocytosis. PLoS Pathog..

[12] Schelhaas M (2012). Entry of human papillomavirus type 16 by actin-dependent, clathrin- and lipid raft-independent endocytosis. PLoS Pathog..

[13] Li E, Stupack D, Bokoch GM, Nemerow GR (1998). Adenovirus endocytosis requires actin cytoskeleton reorganization mediated by Rho family GTPases. J. Virol..

[14] Huang C, Chang SC, Yu IC, Tsay YG, Chang MF (2007). Large hepatitis delta antigen is a novel clathrin adaptor-like protein. J. Virol..

[15] Huang C, Chang SC, Yang HC, Chien CL, Chang MF (2009). Clathrin-mediated post-Golgi membrane trafficking in the morphogenesis of hepatitis delta virus. J. Virol..

[16] Ivanovic T (2011). Recruitment of cellular clathrin to viral factories and disruption of clathrin-dependent trafficking. Traffic..

[17] Legendre-Guillemin V (2005). Huntingtin interacting protein 1 (HIP1) regulates clathrin assembly through direct binding to the regulatory region of the clathrin light chain. J. Biol. Chem..

[18] Ybe JA, Mishra S, Helms S, Nix J (2007). Crystal structure at 2.8 °A of the DLLRKN-containing coiled-coil domain of Huntingtin-interacting protein 1 (HIP1) reveals a surface suitable for clathrin light chain binding. J. Mol. Biol..

[19] Chinchar VG, Waltzek TB (2014). Ranaviruses: not just for frogs. PLoS Pathog..

[20] Chinchar VG, Yu KH, Jancovich JK (2011). The molecular biology of frog virus 3 and other iridoviruses infecting cold-blooded vertebrates. Viruses..

[21] Braunwald J, Nonnenmacher H, Tripier-Darcy F (1985). Ultrastructural and biochemical study of frog virus 3 uptake by BHK-21 cells. J. Gen. Virol..

[22] Qin QW (2001). Electron microscopic observations of a marine fish iridovirus isolated from brown-spotted grouper, *Epinephelus tauvina*. J. Virol. Methods..

[23] Wang S (2014). Entry of a novel marine DNA virus, Singapore grouper iridovirus, into host cells occurs via clathrin-mediated endocytosis and macropinocytosis in a pH-dependent manner. J. Virol..

[24] Chen CY (2002). Clathrin light and heavy chain interface: alpha-helix binding superhelix loops via critical tryptophans. Embo J..

[25] Cureton DK, Massol RH, Saffarian S, Kirchhausen TL, Whelan SPJ (2009). Vesicular Stomatitis Virus Enters Cells through Vesicles Incompletely Coated with Clathrin That Depend upon Actin for Internalization. PLoS Pathog..

[26] Wilbur JD, Chen CY, Manalo V, Hwang PK (2008). Actin binding by Hip1 (huntingtin-interacting protein 1) and Hip1R (Hip1-related protein) is regulated by clathrin light chain. J. Biol. Chem..

[27] Bennett EM, Chen CY, Engqvist-Goldstein AE, Drubin DG, Brodsky FM (2001). Clathrin hub expression dissociates the actin-binding protein Hip1R from coated pits and disrupts their alignment with the actin cytoskeleton. Traffic..

[28] Wakeham DE (2005). Clathrin heavy and light chain isoforms originated by independent mechanisms of gene duplication during chordate evolution. Proc. Natl. Acad. Sci. USA.

[29] Brodsky FM, Chen CY, Knuehl C, Towler MC, Wakeham DE (2001). Biological basket weaving: formation and function of clathrin-coated vesicles. Annu. Rev. Cell Dev. Biol..

[30] Huang XH, Huang YH, Sun J, Han X, Qin QW (2009). Characterization of two grouper *Epinephelus akaara* cell lines: application to studies of Singapore grouper iridovirus (SGIV) propagation and virus-host interaction. Aquaculture..

[31] Huang YH (2011). Transcriptome analysis of orange-spotted grouper (*Epinephelus coioides*) spleen in response to Singapore grouper iridovirus. BMC Genomics..

