# Supplementary material for: The roles of grouper clathrin light chains in regulating the infection of a novel marine DNA virus, Singapore grouper iridovirus
Source: Sci Rep. 2019 Oct 30;9:15647. doi: 10.1038/s41598-019-51725-5 (PMC6821850; doi:10.1038/s41598-019-51725-5)
Supplement: Supplementary file 1 — Supplementary Information [file 41598_2019_51725_MOESM1_ESM.pdf]

Supplementary Information for

The roles of grouper clathrin light chains in regulating the infection of a novel marine DNA virus, Singapore grouper iridovirus

Liqun Wang[1], Qiang Li[2] Songwei Ni[3], Youhua Huang[1], Jingguang Wei[1], Jiaxin Liu[1], Yepin Yu[1], Shaowen Wang[1], Qiwei Qin[1*]**

[1]College of Marine Sciences, South China Agricultural University, Guangzhou, 510642, China.

[2]College of Oceanology and meteorology, Guangdong Ocean University, Zhanjiang, 524088, China.

[3]College of Veterinary Medicine, South China Agricultural University, Guangzhou, 510642, China.

*** Correspondence:** Qiwei Qin: qinqw@scau.edu.cn

****Co-correspondence:** Shaowen Wang**:** wangsw@scau.edu.cn

A

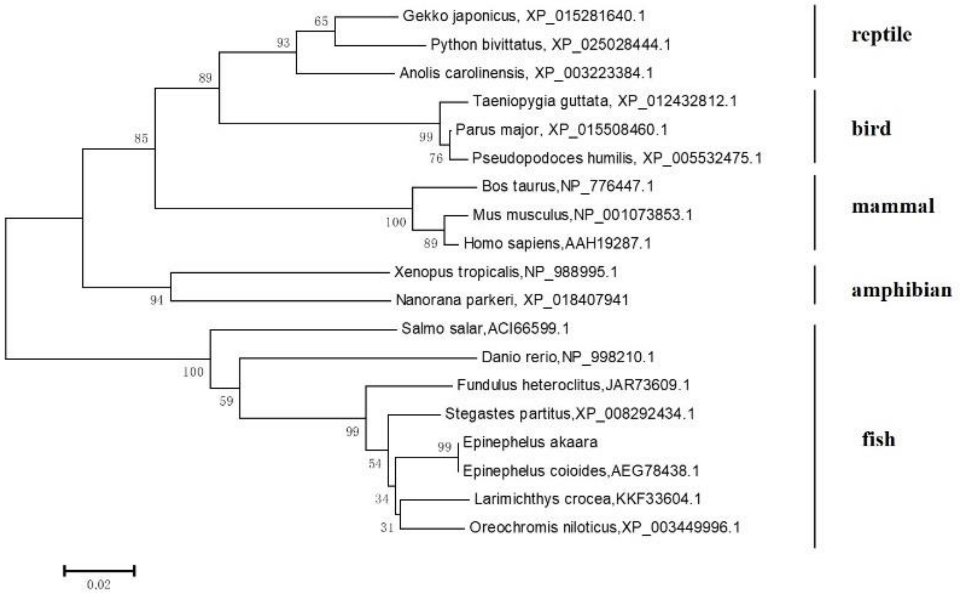

B

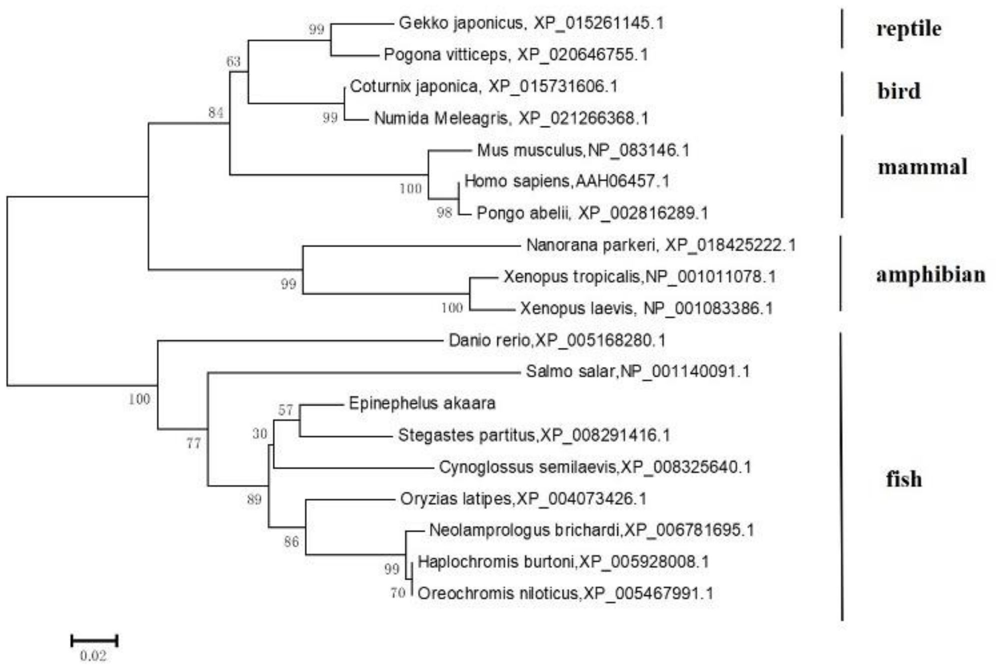

Figure S1. Phylogenetic tree of EaCLCa and EaCLCb with other reported CLCs. The alignment amino acid sequences of CLCs homologs were gained from NCBI database. Phylogenetic tree was composed by the neighbor-joining method within MEGA 4.0. The GenBank accession numbers of selected CLCs sequences were listed on the right of species name

A

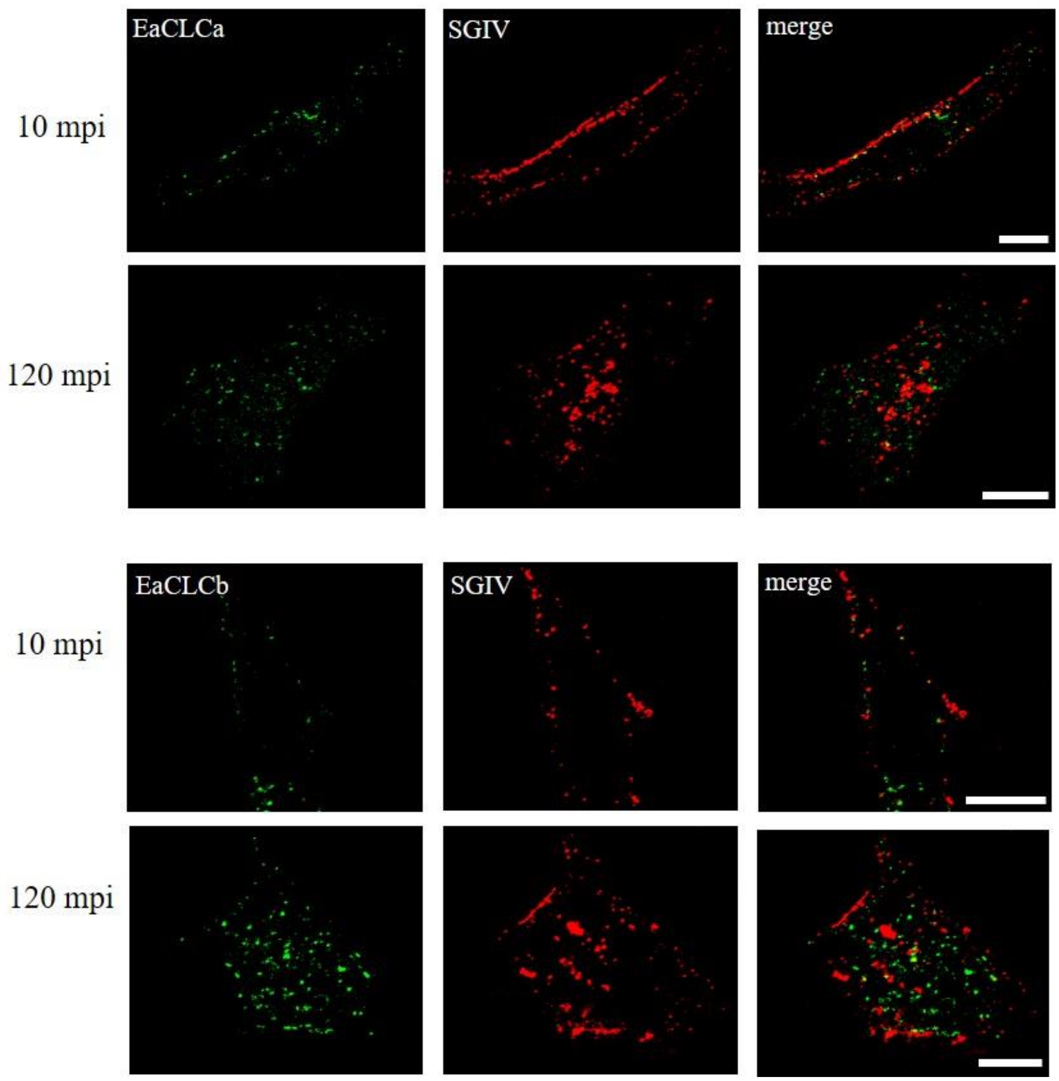

B

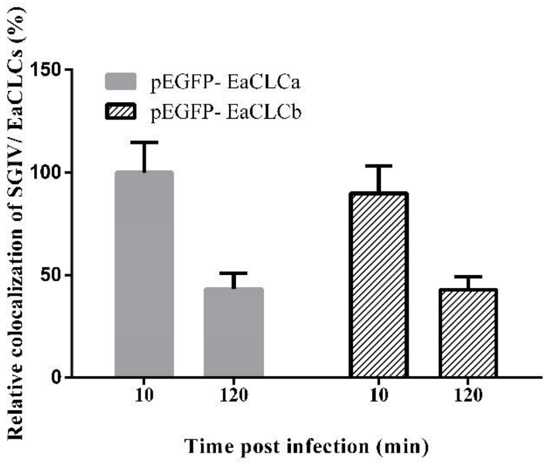

Figure.S2 Colocalization between EaCLCs and SGIV particles changed during SGIV infection. (A) GS cells were transfected with pEGFP-EaCLCa (green) and pEGFP-EaCLCb (green) respectively,

incubated with Alex-Fluor 647 labelled SGIV (red), fixed at different minutes post infection (mpi) and proceeded for confocal imaging. Scale bars indicated 10 μm. (B) Quantification of the colocalization of EaCLCs and SGIV particles. Confocal images were proceeded by MATLAB program for statistical analysis. The colocalization of SGIV and EaCLCs was quantified as the percentage of virus particles colocalized with EaCLCa/EaCLCb relative to the total virus. The colocalization of SGIV and EaCLCa cells was arbitrarily set as 100%.

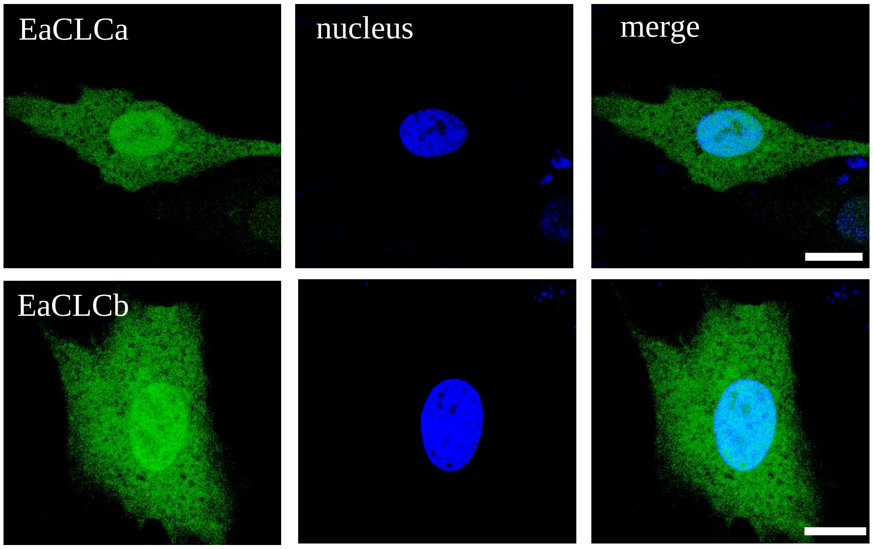

Figure.S3 EaCLCs require bond to CHC for proper localization. GS cells were transfected with pEGFP-EaCLCa-W119R and pEGFP-EaCLCb-W122R respectively, stained by Hoechst33342, and proceeded for confocal imaging. Scale bars indicated 10 μm.

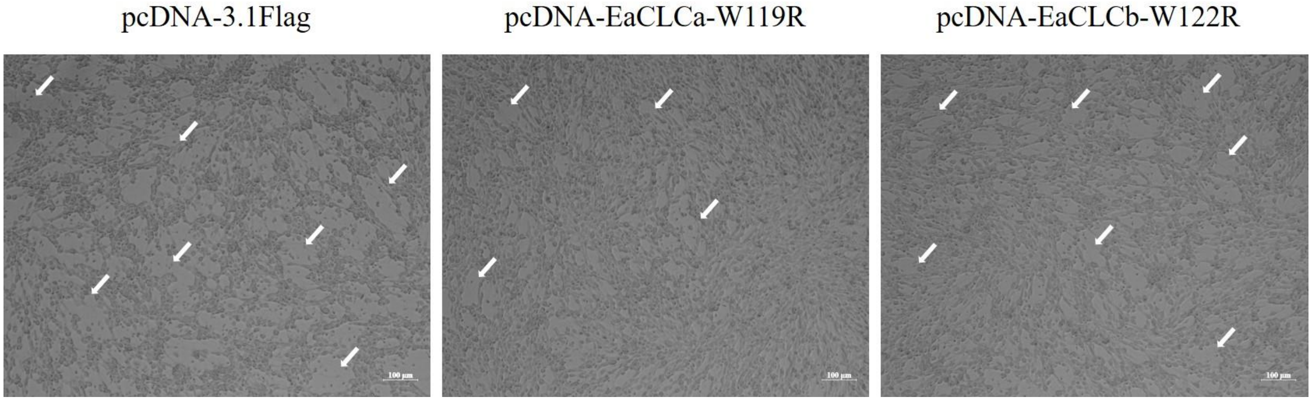

Figure S4. EaCLCa mutant prominently inhibited the process of CPE. Fluorescent microscopy observation of SGIV infection induced CPE in GS cell at 24 hpi. The arrows show the CPE induced by SGIV. Scale bars represent 100 μm.

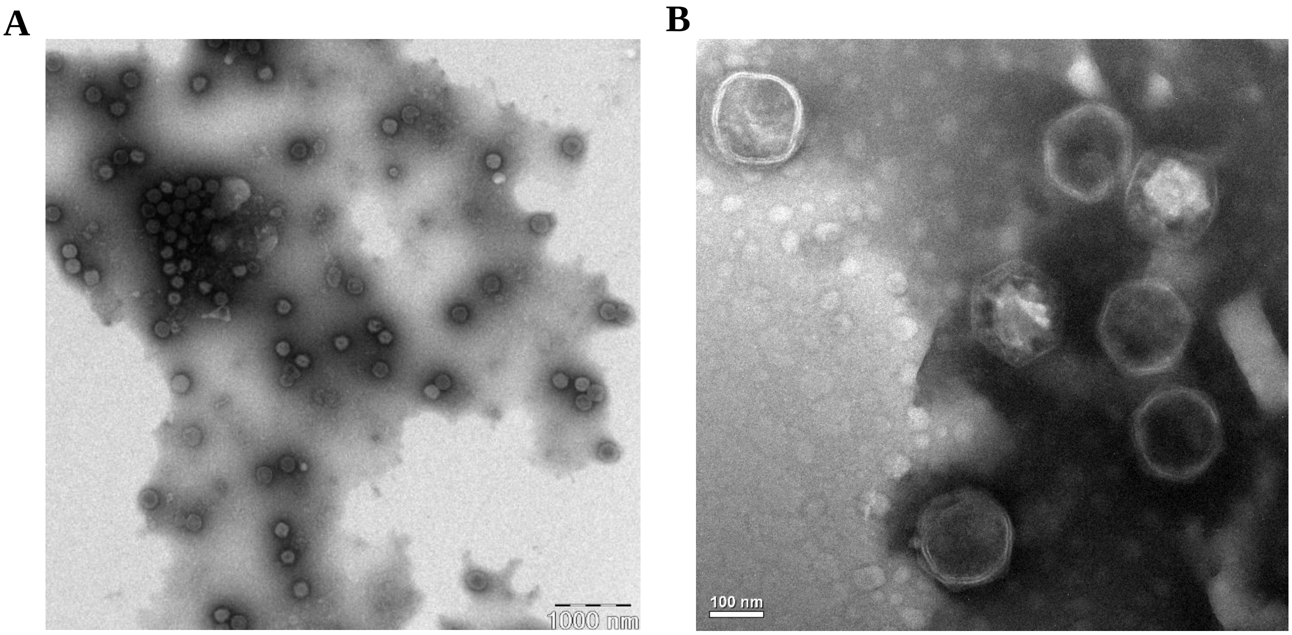

Figure.S5 Transmission electron micrographs of the virus. Electron micrographs of negatively stained the purified SGIV particles (A), and Alex-Fluor 647 labelled SGIV (B).

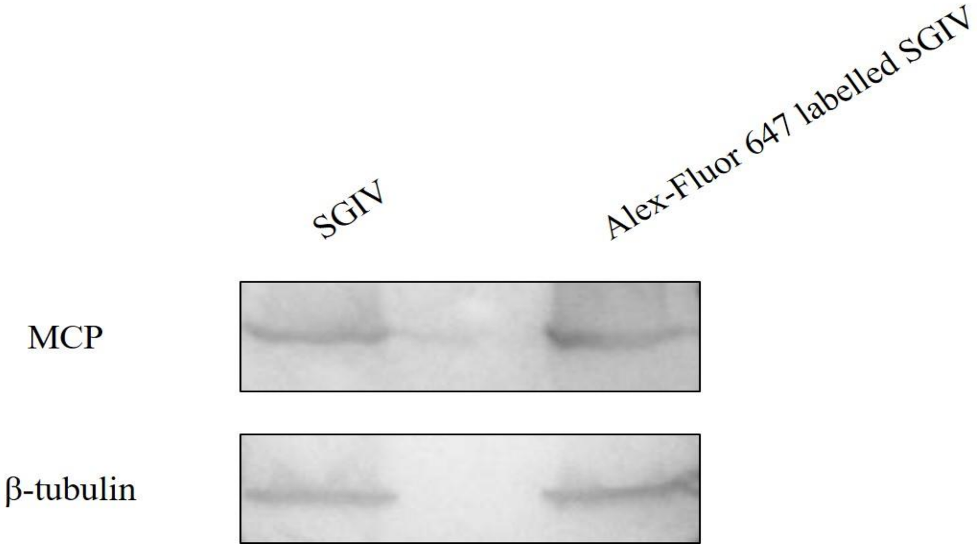

Figure.S6 The protein level of SGIV MCP after infection with SGIV and Alex-Fluor 647 labelled SGIV. GS cells infected with SGIV and Alex-Fluor 647 labelled SGIV, respectively. After 24 h, cells were harvested for western blot, and β-tubulin was used as the internal control.

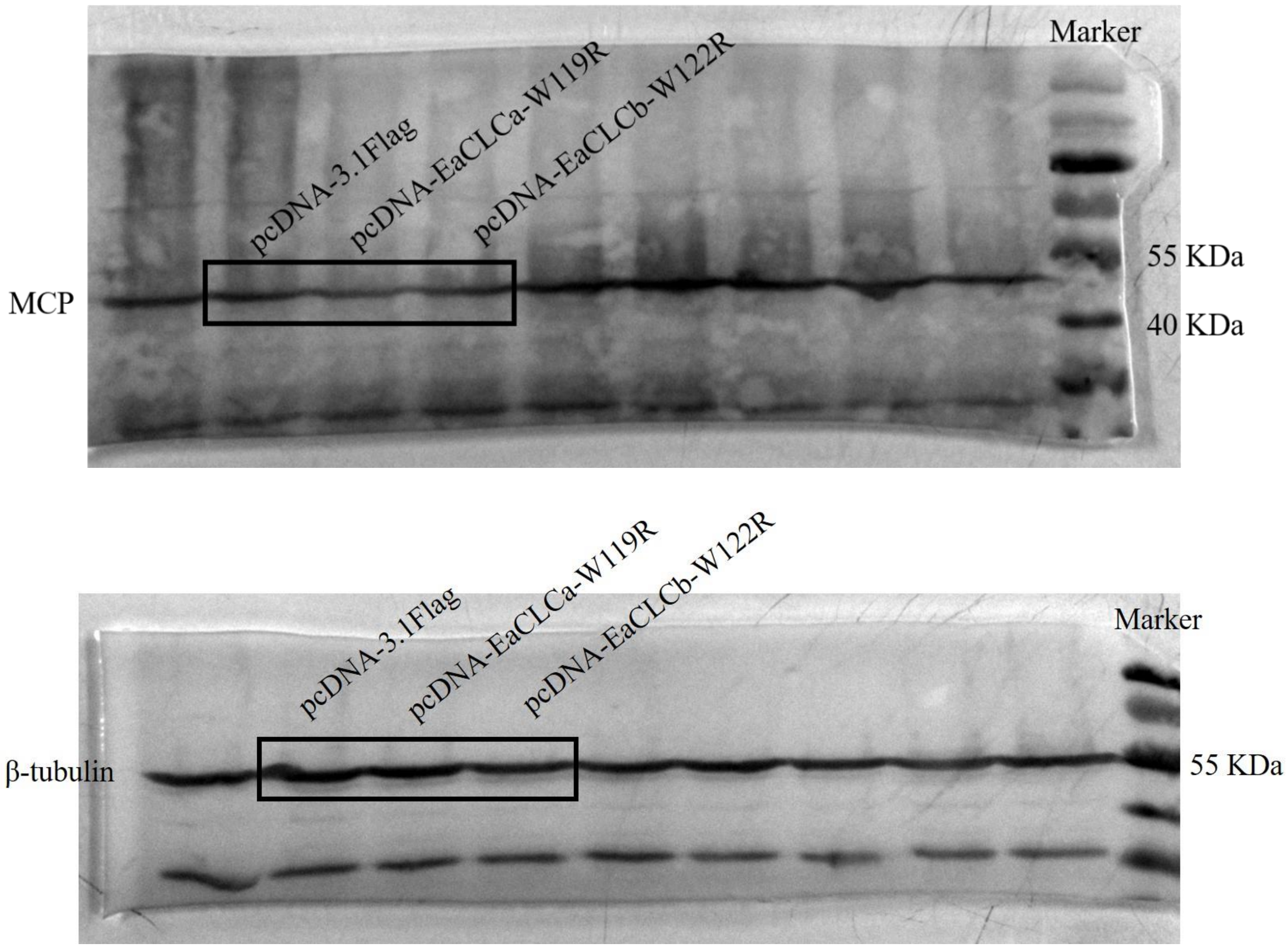

Figure.S7 The image of figure 4B was cropped from the box of this full-length blot.

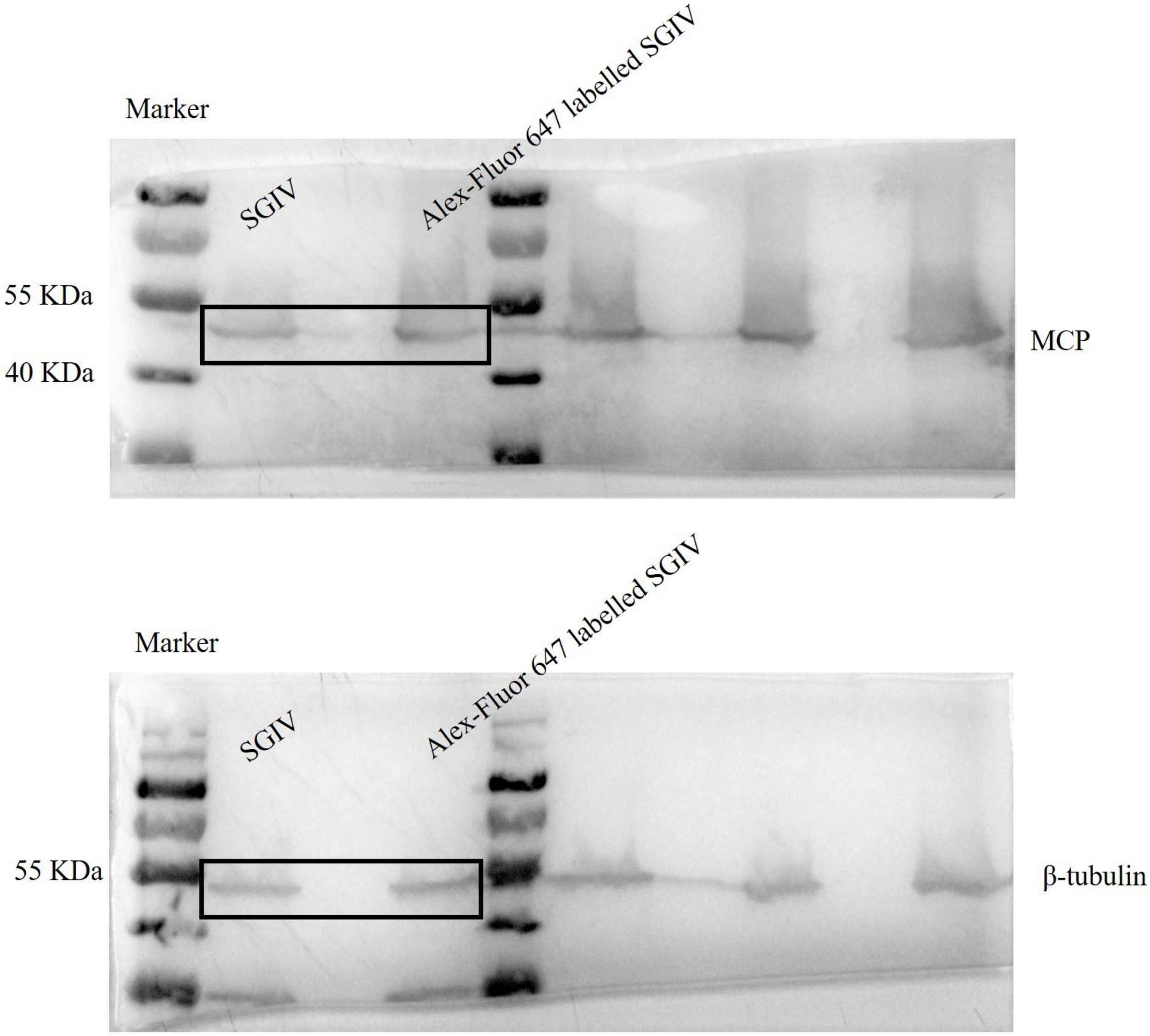

Figure. S8 The image of figure S6 was cropped from the box of this full-length blot.
